# The Genetic Relationship between *Leishmania aethiopica* and *Leishmania tropica* Revealed by Comparing Microsatellite Profiles

**DOI:** 10.1371/journal.pone.0131227

**Published:** 2015-07-21

**Authors:** Lena Krayter, Lionel F. Schnur, Gabriele Schönian

**Affiliations:** 1 Institute of Microbiology and Hygiene, Charité-University Medicine, Berlin, Germany; 2 Department of Parasitology, The Kuvin Center for the Study of Infectious and Tropical Diseases, Hebrew University-Hadassah Medical School, Jerusalem, Israel; Wuhan Botanical Garden, Chinese Academy of Sciences, Wuhan, China, CHINA

## Abstract

**Background:**

*Leishmania* (*Leishmania*) *aethiopica* and *L*. (*L*.) *tropica* cause cutaneous leishmaniases and appear to be related. *L*. *aethiopica* is geographically restricted to Ethiopia and Kenya; *L*. *tropica* is widely dispersed from the Eastern Mediterranean, through the Middle East into eastern India and in north, east and south Africa. Their phylogenetic inter-relationship is only partially revealed. Some studies indicate a close relationship. Here, eight strains of *L*. *aethiopica* were characterized genetically and compared with 156 strains of *L*. *tropica* from most of the latter species' geographical range to discern the closeness.

**Methodology/Principal Findings:**

Twelve unlinked microsatellite markers previously used to genotype strains of *L*. *tropica* were successfully applied to the eight strains of *L*. *aethiopica* and their microsatellite profiles were compared to those of 156 strains of *L*. *tropica* from various geographical locations that were isolated from human cases of cutaneous and visceral leishmaniasis, hyraxes and sand fly vectors. All the microsatellite profiles were subjected to various analytical algorithms: Bayesian statistics, distance-based and factorial correspondence analysis, revealing: (i) the species *L*. *aethiopica*, though geographically restricted, is genetically very heterogeneous; (ii) the strains of *L*. *aethiopica* formed a distinct genetic cluster; and (iii) strains of *L*. *aethiopica* are closely related to strains of *L*. *tropica* and more so to the African ones, although, by factorial correspondence analysis, clearly separate from them.

**Conclusions/Significance:**

The successful application of the 12 microsatellite markers, originally considered species-specific for the species *L*. *tropica*, to strains of *L*. *aethiopica* confirmed the close relationship between these two species. The Bayesian and distance-based methods clustered the strains of *L*. *aethiopica* among African strains of *L*. *tropica*, while the factorial correspondence analysis indicated a clear separation between the two species. There was no correlation between microsatellite profiles of the eight strains of *L*. *aethiopica* and the type of leishmaniasis, localized (LCL) versus diffuse cutaneous leishmaniasis (DCL), displayed by the human cases.

## Introduction

Four species of *Leishmania* are known to cause cutaneous leishmaniasis (CL) in Ethiopia: *L*. *major*, *L*. *tropica*, *L*. *aethiopica* and, in some cases, *L*. *donovani*, which mainly causes visceral leishmaniasis (VL) but, also, cutaneous lesions without patent signs and symptoms of visceral infection and, also, in some cases, post-kala-azar dermal leishmaniasis (PKDL) as a cutaneous sequel, following the chemotherapeutic treatment of VL. Of these four leishmanial species, only *L*. *aethiopica* is restricted to Africa and even there, as far as has been recorded, just to the highlands of Ethiopia and Kenya. In Djibouti and Eritrea cases of CL have been reported, but the causative species has not been determined. This restricted distribution is, probably, owing to the restricted geographical range of the sand fly vector species *Phlebotomus longipes* and *P*. *pedifer* that transmit *L*. *aethiopica* among their animal reservoirs and humans [1,2]. A strain of *L*. *aethiopica*, which proved to be the type strain of a new zymodeme of the species, was isolated from a female sand fly of the species *P*. *sergenti* caught in Awash Valley, north-eastern Ethiopia, in the 1990s [3] and Alvar *et al*. have also listed *P*. *sergenti* as a vector of *L*. *aethiopica* together with *P*. *longipes* and *P*. *pedifer* [2]. They have also listed *P*. *pedifer* and *P*. *aculeatus* (also named *P*. *elgonensis in some articles)* as the vectors of *L*. *aethiopica* in Kenya [2]. However, some taxonomists working on sand flies maintain that the female sand flies of the species *P*. *pedifer* are morphologically indistinguishable from the non-vector *P*. *aculeatus* although they can be distinguished by isoenzyme analysis. In Ethiopia, the animal reservoirs are hyraxes of the species *Procavia capensis and Heterohyrax brucei* [1,2]. In Israel and Namibia, hyraxes of the species *Procavia capensis* have also been incriminated as reservoir hosts of *L*. *tropica* [4–8]. In Kenya, the giant pouch rat *Cricetomys sp*. has been suggested as serving as reservoir host of *L*. *aethiopica* in addition to hyraxes [2].

In Ethiopia, human cases of leishmaniasis caused by *L*. *aethiopica* occur as one of three possible clinical conditions: localized cutaneous leishmaniasis (LCL); diffuse cutaneous leishmaniasis (DCL); and, rarely, muco-cutaneous leishmaniasis (MCL). In Kenya, only cases of LCL have been recorded [2].

When strains of *L*. *aethiopica* were first isolated from human cases of LCL and DCL, they were considered to be strains of *L*. *tropica*. However, owing to various criteria they were described and named a new species, *L*. *aethiopica*: the restricted geography of human cases and the association of some of the strains with DCL, a condition, at that time, only known from the New World; the specificity of the sand fly vectors; the animal reservoir hosts being hyraxes, which was unusual at that time; and the antigenic specificity of the strains shown by serotyping using indirect haemagglutination[9]. The antigenic specificity of strains of *L*. *aethiopica* was later corroborated by excreted factor (EF) serotyping [10]. Many of the Ethiopian and Kenyan strains of *L*. *aethiopica* collected over the past 30 to 40 years have been serotyped according to the type of EF they produced. All those examined were of the EF sub-serotype B_1_, a sub-serotype that has, so far, been unique to the species *L*. *aethiopica*, separating its strains antigenically from those of all the other Old and New World species of *Leishmania*.

However, some phylogenetic studies based on targeting different cellular components of the parasites have indicated that *L*. *aethiopica* has a closer relationship to either *L*. *tropica* or *L*. *major*. For example, several studies based on the electrophoretic mobilities of enzymes (MLEE) exposed a closer relationship between *L*. *aethiopica* and *L*. *tropica* on comparing the enzyme profiles of various leishmanial strains [5,11]. However, other MLEE studies based on the mobility profiles of other enzymes exposed a closer relationship between strains of *L*. *aethiopica* and *L*. *major* with them forming a monophyletic group [12–14]. Sequencing of a 370 bp fragment of the coding region of the heat shock protein gene *hsp*20 supported a closer relationship between *L*. *aethiopica* and *L*. *major* [15]. A further hint for the close relationship between *L*. *aethiopica* and *L*. *tropica*, although not statistically supported, is the disability to distinguish between these two species by RFLP of the *hsp*70 gene with the restriction enzyme *Hae*III [16]. A further enzyme, *Bsa*HI is required to discriminate between these two species [16]. The need for the second restriction enzyme *Bsa*HI was also the case in distinguishing between the species *L*. *donovani* and *L*. *infantum*, which both belong to the *L*. *donovani* species complex. A study combining internal transcribed spacer 1*-Restriction Fragment Length Polymorphism* (ITS1-RFLP), using ten different restriction enzymes together with DNA fingerprinting combined the analysed strains of *L*. *aethiopica* and *L*. *major* into a monophyletic group, [17]. In reverse line blot hybridisation, two of three probes developed for *L*. *tropica* also gave positive signals for *L*. *aethiopica*, indicating genetic similarities in the DNA of strains of these two species [18]. None of the other probes prepared for that study gave positive results using the DNA of any other leishmanial species. Finally, a Multilocus Sequence Analysis (MLSA) of 552 polymorphic sites on a 4,677bp-long concatenated sequence spread over seven single copy DNA coding sequences located on six different chromosomes produced a monophyletic group consisting of strains of *L*. *aethiopica* and strains belonging to the *L*. *tropica* complex [19].

Although the phylogenetic inter-relationship of the species *L*. *aethiopica*, *L*. *tropica* and *L*. *major* differs, depending on the methods used to differentiate strains, all the above methods and many others like ITS1-RFLP [20], High Resolution Melt Analysis (HRM) [21] and PCR approaches using species-specific primers [22,23] clearly separate *L*. *aethiopica* from the other species. Kebede *et al*. [23] developed five microsatellite markers for typing and identifying strains of *L*. *aethiopica*, a battery considered too small to adequately determine precise genetic relationships among strains of *Leishmania*.

Multilocus microsatellite typing (MLMT) has been used successfully to reveal intra-species phylogenetic relationships [7,24–26] but have not been applied to revealing inter-species relationships except for strains of *L*. *infantum* and *L*. *donovani*, the two species forming the *L*. *donovani* species complex [24,27]. Although single microsatellite markers can be amplified in several different species, there are always markers in a set that are species-specific and fail to get amplified in other species. In the set of 12 microsatellite markers prepared previously to type strains of *L*. *tropica* [28,29], four (LIST7010, LIST7011, 4GTG, 27GTG) were originally developed for genotyping strains of *L*. *major* [25,30], four (LIST7027, LIST7033, LIST7039, LIST7040) for genotyping strains of *L*. *donovani* [31] and four (GA1, GA2, GA6, GA9) for genotyping *L*. *tropica* [32]. Since the species *L*. *aethiopica* and *L*. *tropica* have been shown to display a high degree of genetic similarity, the 12 microsatellite markers that were used to genotype strains of *L*. *tropica* were applied in this study to genotype strains of *L*. *aethiopica* isolated from eight human cases of CL; and by using the same set of microsatellite markers on strains from both species, a phylogenetic analysis was enabled, based on comparing and combining the microsatellite profiles encompassed by the species *L*. *aethiopica* and *L*. *tropica* that attempted to determine their genetic inter-relationship.

## Methods

### Ethical clearance

Only previously gathered and cryopreserved strains have been used in this study. All the strains of *L*. *aethiopica* were isolated from human cases by needle and syringe aspiration during routine diagnosis with no other invasive procedures at the time of clinical examination. Cases' personal data were coded for anonymity when samples were collected. Strain collection and Leishmania DNA used in this study were described in previous publications [17,33].

### Parasite strains

Eight strains of *L*. *aethiopica* were typed that came from different Ethiopian foci of CL between 1972 and 1994. They were isolated from human cases, who presented with either LCL or DCL at local hospitals. Their WHO codes are given in S1 Table. Owing to their genetic similarity to strains of *L*. *tropica*, 156 strains of *L*. *tropica*, whose microsatellite profiles were published previously [7,28], were included for a global analysis. The strain of *L*. *tropica* MHOM/PS/2001/ISL590, whose microsatellite loci have been sequenced [32], was used as a reference strain to compare the results of different PCR and fragment analysis runs.

### Microsatellite typing

The set of twelve independent unlinked microsatellite markers used previously for population genetic studies of *L*. *tropica* [28,29] were used here for strains of species *L*. *aethiopica* and *L*. *tropica*. The microsatellite markers were amplified using labelled primers, as previously described [28], and the lengths of the resulting fragments were determined with an ABI sequencer. GeneMapper software version 3.7 (Applied Biosystems, Foster City, USA) was applied for analysis of the raw data. A single peak in the GeneMapper was assumed to represent two identical alleles at the specific locus; two peaks indicated a heterozygous locus. In the latter case, both fragment lengths were used for the analyses. All detected fragments are listed in S1 Table. Strain MHOM/PS/2001/ISL590 was included in each run as a standard for fragment size.

### Population genetic analysis

The input file compiled in an excel table and saved as text file was converted with MSA version 4.05 [34]. STRUCTURE software version 2.3.4 [35] implements a Bayesian clustering approach and was used to investigate the population structure. This approach determines genetically distinct groups based on allele frequencies and estimates the group membership of each individual. The length of the Burnin Period was set to 20000 and the number of Markov Chain Monte Carlo Repeats after Burnin to 200000. DeltaK (ΔK) calculation, as described in [36], revealed the most probable number of populations, using the results from Bayesian statistics for ten replicate runs for each K.

Factorial Correspondence Analysis (FCA) as implemented in Genetix 4.05 [37] was applied to reveal the genetic distances between the single populations in a three-dimensional space according to their allelic state similarities. The input files for Genetix were converted using CONVERT 1.31 [38]. Using POPULATIONS software version 1.2.34 (http://bioinformatics.org/~tryphon/populations/) genetic distances were calculated based on Chord distances. The resulting Neighbour Joining (NJ) tree was displayed with MEGA 5.1 [39]. SplitsTree software version 4.12.8 [40] was applied to create a phylogenetic network revealing reticulation events between certain populations. The genetic distances between populations (*F*_ST_ values) were calculated using MSA 4.05. The mean number of alleles (A), observed (*H*_o_) and expected (*H*_e_) heterozygosity and the inbreeding coefficients (*F*_IS_) were determined using GDA 1.1[41].

## Results

Six of the Ethiopian strains had been identified as *L*. *aethiopica* by ITS1-RFLP analysis previously [17] and two, MHOM/ET/1972/L100 (= LRC-L147) and MHOM/ET/1985/LRC-L494, were identified as such by ITS1-RFLP analysis during this study. The strain MHOM/ET/1972/L100 (= LRC-L147) came from a case of DCL recorded as coming from near the City of Nekemte, Wolega Province, northern Ethiopia. The strain MHOM/ET/1985/LRC-L494 was isolated in Israel from an Ethiopian immigrant with CL. The location in Ethiopia where he acquired his CL was not recorded (S1 Table).

Amplification by a PCR and the subsequent fragment analysis of the microsatellites in the DNA samples from the eight strains of *L*. *aethiopica* (S1 Table) succeeded, using the set of markers prepared for and applied to microsatellite typing of strains of *L*. *tropica* and population genetics of the species *L*. *tropica* [28]. This, in itself, pointed to a fairly close genetic relationship between *L*. *aethiopica* and *L*. *tropica*. Some of the fragment sizes determined were identical to those previously found in strains of *L*. *tropica* although others seemed to be unique to strains of *L*. *aethiopica* (S1 Table). Each one of the eight strains of *L*. *aethiopica* analysed had a unique microsatellite profile, i. e., profiles *Laet*MS 001, -002, -003, -004, -005, -006, -007, and -008. Heterozygous alleles comprised either 27.1% or 29.8% of all the alleles, respectively, depending on whether markers that failed to be amplified were considered as being homozygous or were excluded from the calculation.

In this population genetic study, 164 strains of *Leishmania*, eight of *L*. *aethiopica* from Ethiopia and 156 of *L*. *tropica* from a wide range of geographical origins, were analysed (S1 Table). The microsatellite profiles of the latter have been published previously [7,28] The 164 strains of *Leishmania* examined yielded 101 different microsatellite profiles, of which 79 were encountered in just one strain and 85 were shared by more than one strain. The number of different alleles for the 12 loci ranged from three (microsatellite markers GA6 and GA9n) to 17 (microsatellite marker LIST7039) with an average of 8.75 (Table 1). The mean number of alleles per population was 2.2 (Table 2). The sub-population consisting of the strains of *L*. *aethiopica* showed the highest mean number of alleles (3.25) despite the low number of individuals (n = 8). The observed heterozygosities were lower than the expected heterozygosities in all (sub-)populations except for the sub-population Sanliurfa, Turkey, thus leading to positive *F*_IS_ values. The population containing the strains of *L*. *aethiopica* had a *F*_IS_ value of 0.492.

**Table 1:** Descriptive statistics per locus.

locus	A	*H*_o_	*H*_e_	*F*_IS_
**GA1**	6	0.024	0.129	0.811
**GA2**	10	0.079	0.694	0.886
**GA6**	3	0.000	0.161	1.000
**GA9n**	3	0.354	0.513	0.311
**LIST7010**	11	0.157	0.824	0.810
**LIST7011**	10	0.019	0.745	0.975
**LIST7027**	13	0.378	0.820	0.540
**LIST7033**	11	0.037	0.615	0.940
**LIST7039**	17	0.119	0.795	0.851
**LIST7040**	11	0.323	0.673	0.521
**4GTG**	4	0.018	0.272	0.933
**27GTGn**	6	0.188	0.667	0.719
**all**	**8.75**	**0.141**	**0.576**	**0.755**

A, number of alleles; *H*_o_, observed heterozygosity; *H*_e_, expected heterozygosity; *F*_IS_, inbreeding coefficient (-1 = outcrossing, 0 = random mating, +1 = inbreeding)

**Table 2:** Descriptive statistics per population.

population	A	*H*_o_	*H*_e_	*F*_IS_
***L*. *aethiopica***	3.250	0.266	0.507	0.492
**Kenya/Tunisia**	2.833	0.097	0.526	0.829
**Namibia/Kenya**	1.750	0.021	0.302	0.942
**MoroccoA/Turkey**	1.833	0.042	0.298	0.867
**MoroccoB**	1.750	0.125	0.286	0.633
**Northern Galilee**	1.833	0.025	0.226	0.895
**Sanliurfa, TR**	1.417	0.318	0.176	-0.849
**old strains/Palestine**	2.917	0.328	0.359	0.087
**Israel/Palestine**	2.417	0.030	0.140	0.788
**mean**	**2.222**	**0.139**	**0.313**	**0.563**

A, number of alleles; *H*_o_, observed heterozygosity; *H*_e_, expected heterozygosity; *F*_IS_, inbreeding coefficient (-1 = outcrossing, 0 = random mating, +1 = inbreeding)

Bayesian clustering as implemented in STRUCTURE and calculation of deltaK values (S1 Fig) assigned the strains to three main populations. All eight strains of *L*. *aethiopica* clustered together with 20 African strains of *L*. *tropica* from Morocco, Tunisia, Kenya and Namibia, ten Israeli ones from human cases of CL and sand fly vectors from a focus north of the Sea of Galilee [8] and two Turkish ones from human cases of CL as one main population designated Africa/Galilee (S1 Table). The other two main populations were designated Turkey/old strains/Palestine and Israel/Palestine, also according to the geographical origins of the strains they comprised (S1 Table). The population Turkey/old strains/Palestine included 24 strains collected in 1995 during a localized outbreak of CL at Sanliurfa, south-eastern Turkey, 27 of various origins collected between 1949 and 1999, and six Palestinian ones collected in 2002. Some of the strains of *L*. *tropica* from Morocco, Turkey and Namibia assigned to the population African/Galilee also showed shared membership in the population Turkey/old strains/Palestine (S2 Fig). The population Israel/Palestine comprised 66 strains from these two areas and one strain from the Sinai Peninsula, Egypt.

**Fig 1:**
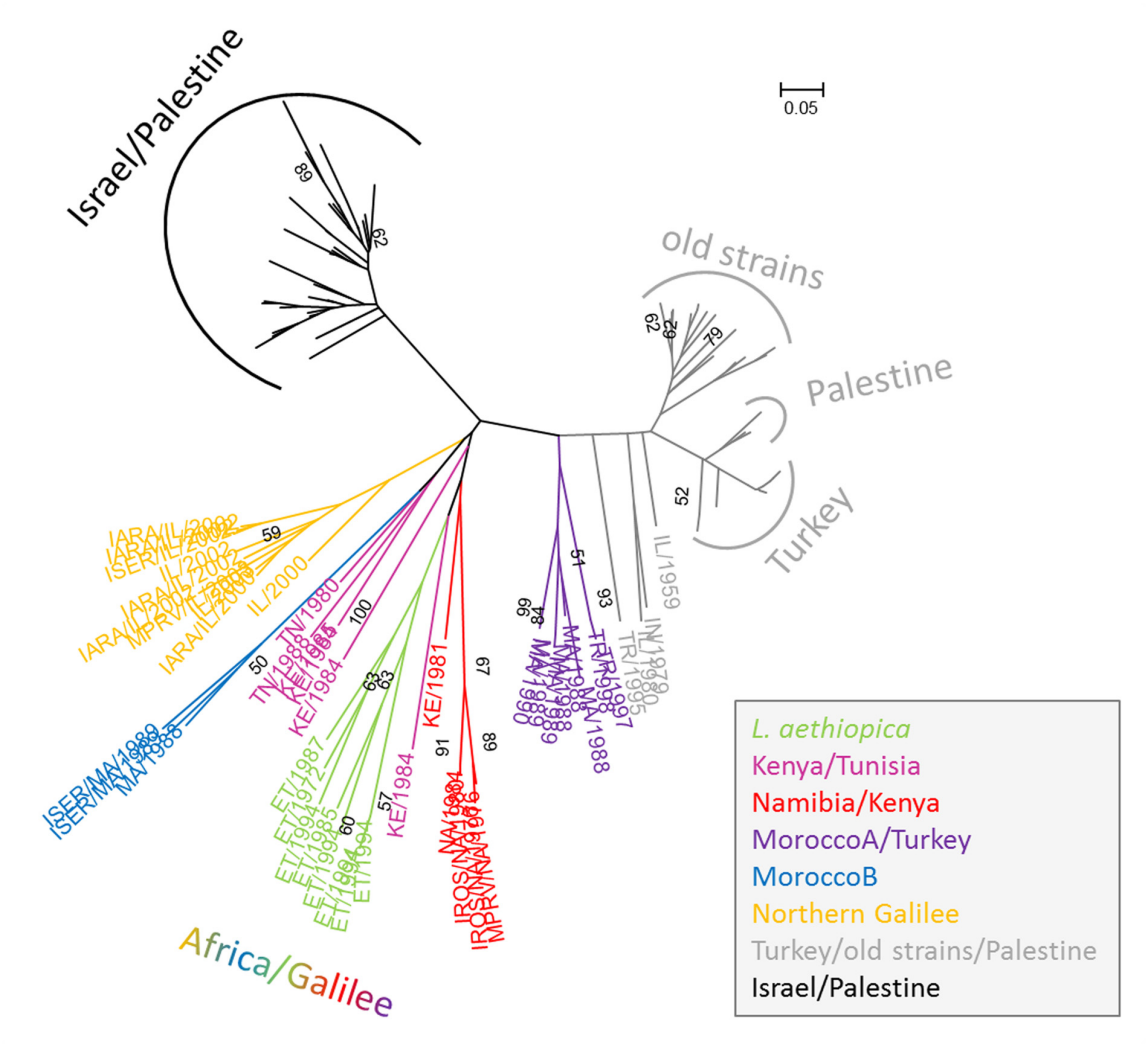
Neighbour Joining tree displaying the phylogenetic relationship between strains of *L*. *aethiopica* and *L*. *tropica*. Bootstrap values >50 are indicated at the nodes. For clarity, only the WHO codes of the strains in population Africa/Galilee and of the four ' intermediate ' strains IL/1959, IN/1979, IL/1980, TR/1995 are given. The partial WHO codes specify the host, the country of origin and the year of isolation: I = insect, SER = *P*. *sergenti*, ARA = *P*. *arabicus*, ROS = *P*. *rossi*, M = mammal, PRV = *Procavia* (hyrax). If not indicated otherwise, the strains were isolated from human cases. The full WHO codes of all the strains are given in S1 Table. The different colours indicate the clustering based on the Bayesian statistical results at the sub-population level for the population Africa/Galilee; the main populations Turkey/old strains/Palestine and Israel/Palestine are in grey and black, respectively.

**Fig 2:**
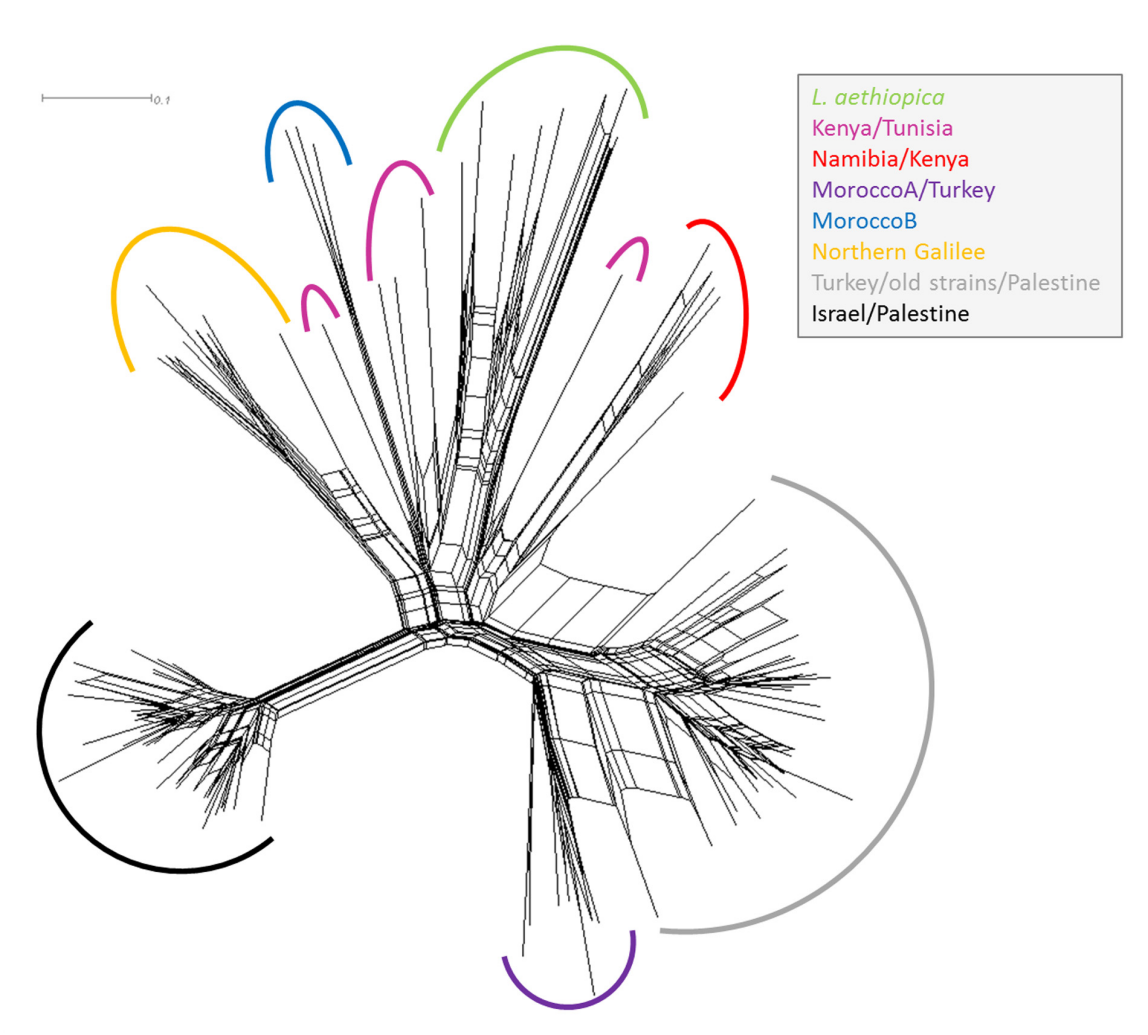
Network presenting the genetic relationship between strains of *L*. *aethiopica* and *L*. *tropica*. Cross connections indicate probable reticulation events like hybridisation, recombination and horizontal gene transfer between the strains. The sub-populations within the population Africa/Galilee resulting from clustering based on Bayesian statistical results are in different colours; the main populations Turkey/old strains/Palestine and Israel/Palestine are highlighted in grey and black, respectively.

**Fig 3:**
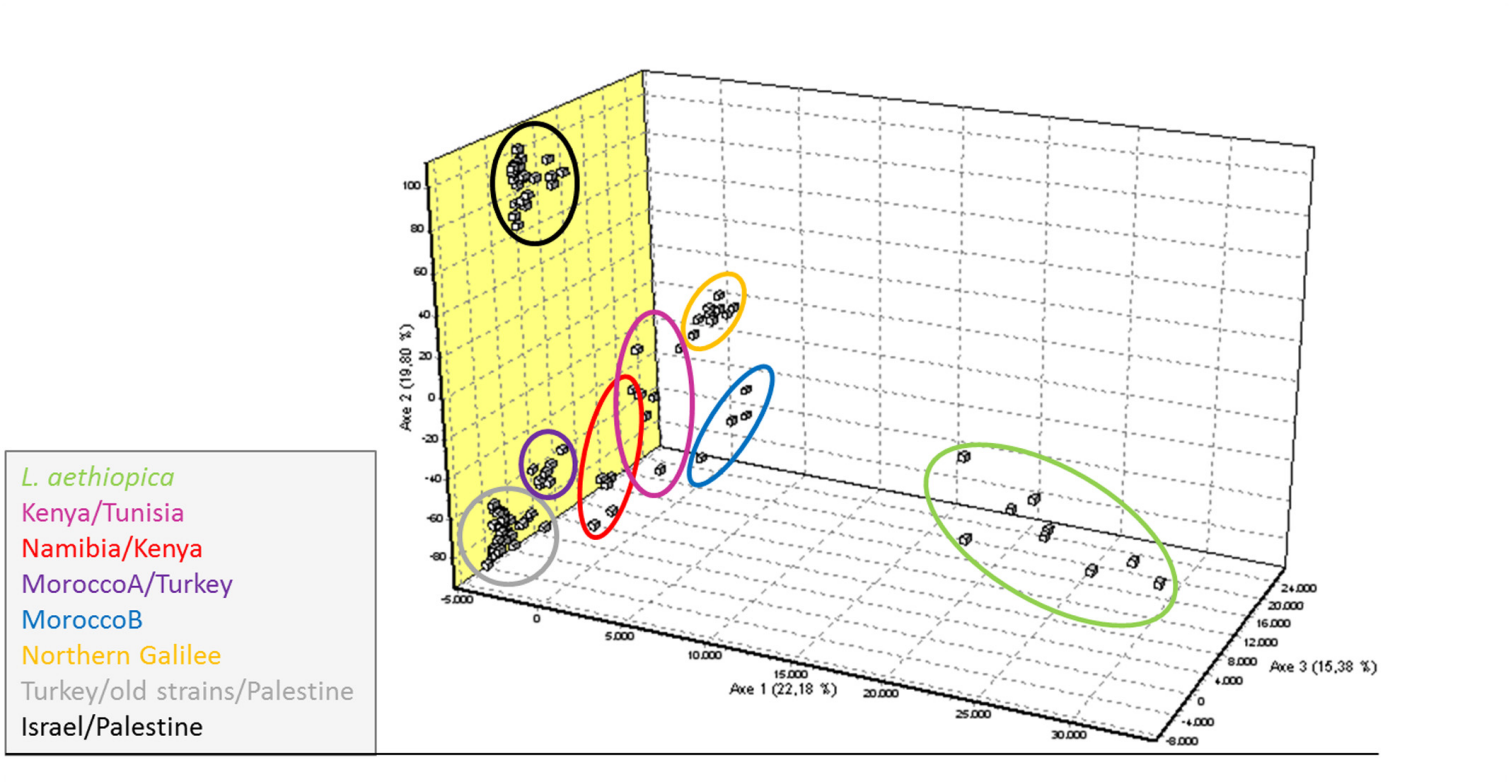
Factorial correspondence analysis (FCA) of the nine sub-populations. FCA displays the calculated genetic distances 3-dimensionally based on allele similarities. Each square represents one genotype. The colours correspond to clustering based on Bayesian statistical results. The software requires pre-assignment to single populations, therefore strains were assigned to the nine sub-populations proposed by STRUCTURE.

The existence of these three main populations was broadly confirmed by the applied distance based population genetic methods shown in Figs 1 and 2. A group of six Moroccan and two Turkish strains of *L*. *tropica* previously assigned to the population Africa/Galilee by STRUCTURE was found to be more closely related to the population Turkey/old strains/Palestine by the genetic distance analyses, which was confirmed by the FCA (Fig 3).

To reveal the phylogenetic position of the strains of *L*. *aethiopica* relative to those of *L*. *tropica*, the main population Africa/Galilee was scrutinized. Re-analysis by the Bayesian clustering approach revealed three sub-populations according to deltaK estimation even though the strength of the signal was quite low (S1 Fig). Evaluating the 10 iterations produced by STRUCTURE gave no definite assignment of the strains to these three groups. However, a second peak in the deltaK calculation proposed six groups within the population Africa/Galilee but, again, the signal was weak. However, this division into six groups was broadly consistent in all ten iterations made and the strains of *L*. *aethiopica* formed a separate cluster in seven out of ten iterations. Three sub-structures were stable in all ten iterations: sub-population Namibia/Kenya, containing four strains from Namibia and one from Kenya; sub-population MoroccoA/Turkey, containing six strains from Morocco and two from Turkey; and sub-population Northern Galilee, containing all ten strains from the focus on the northern side of the Sea of Galilee. Three other strains from Morocco, sub-population MoroccoB, clustered together in all ten iterations, forming a separate sub-population in only eight out of ten iterations. Another four strains from Kenya and two from Tunisia clustered together in eight out of ten iterations, sub-population Kenya/Tunisia. Of those strains sharing membership of both the population Africa/Galilee and the population Turkey/old strains/Palestine, the Moroccan and Turkish ones constituted the sub-population MoroccoA/Turkey and the Namibian ones were part of the sub-population Namibia/Kenya. The colours in Figs 1–3 and in S1 Table indicate the six sub-populations.

Regarding the strains of *L*. *tropica*, the NJ tree (Fig 1) confirmed the populations Israel/Palestine and Turkey/old strains/Palestine as two quite distinct entities as inferred by the Bayesian clustering approach. However, the African strains and those from the focus north of the Sea of Galilee did not form a distinct population. Those from Morocco and Turkey that had formed the sub-population MoroccoA/Turkey by the STRUCTURE analysis were now completely separate from all the other African strains and closer to the population Turkey/old strains/Palestine. The branches comprising the African strains and those from north of the Sea of Galilee diverged close to the origin of that main branch of the tree and the resulting long branches indicate relatively broad genetic diversification among these strains. However, their separation into sub-populations in the NJ tree resembled that according to the Bayesian clustering. The strains of *L*. *aethiopica* formed one monophyletic group that was most closely related to the strains of *L*. *tropica* from the sub-population Namibia/Kenya. The strains of *L*. *tropica* from north of the Sea of Galilee and those of the sub-population MoroccoB clustered as separate monophyletic groups. In contrast to the Bayesian results, the strains of the sub-population Kenya/Tunisia did not form a distinct cluster but were dispersed among other strains in this main branch of the tree.

The network analysis produced the same arrangement of clusters seen in the NJ tree (compare Figs 1 and 2), confirming the sub-populations Namibia/Kenya, MoroccoA/Turkey, MoroccoB, Northern Galilee and the separate clustering of the strains of *L*. *aethiopica*, and also the dispersed nature of the remaining strains from Kenya and Tunisia. Again, the group MoroccoA/Turkey was found to be more closely related to the main population Turkey/old strains/Palestine but now located on the very edge of that population. The many cross connections within the cluster containing the strains of *L*. *aethiopica* probably indicates the occurrence of reticulation events like hybridisation, recombination and horizontal gene transfer between the strains.

FCA analysis was applied to the nine (sub-)populations of strains derived by Bayesian clustering: Israel/Palestine; Sanliurfa, Turkey; old strains/Palestine; Kenya/Tunisia; Namibia/Kenya; MoroccoA/Turkey; MoroccoB; Northern Galilee; and *L*. *aethiopica*. Interestingly, this approach placed the strains of *L*. *aethiopica* distantly from all the strains of *L*. *tropica* (Fig 3). The sub-populations *L*. *aethiopica*, MoroccoA/Turkey, MoroccoB and Northern Galilee exposed by the other analytical methods were confirmed. The sub-populations containing the Kenyan strains, sub-populations Kenya/Tunisia and Namibia/Kenya, could not be clearly separated by this approach. Here, one Kenyan strain from each of the sub-populations Kenya/Tunisia and Namibia/Kenya clustered in the other sub-population. When considering only the African strains and excluding the two other main populations Israel/Palestine and Turkey/old strains/Palestine from the data set, the separation of the sub-populations Kenya/Tunisia and Namibia/Kenya improved but one strain from the sub-population Namibia/Kenya still fell closer to the strains in the sub-population Kenya/Tunisia (S3 Fig). The sub-populations Sanliurfa, Turkey and old strains/Palestine could not be confirmed by this method; their strains were indistinguishable.

*F*_ST_ estimates indicate the genetic distances between the distinct populations. All the estimates revealed exceedingly high genetic differentiation in each pairwise comparison (S2 Table). Some of the comparisons were not significant though the differentiation was categorized as very great (>0.25). Some of the fixation indices were classified as insignificant by MSA software (S2 Table). This resulted from the low number of strains in particular populations: only 11 in the pairwise comparisons between the sub-populations MoroccoB and *L*. *aethiopica* (*F*_ST_ = 0.520), MoroccoB and MoroccoA/Turkey (*F*_ST_ = 0.610), and Namibia/Kenya and Kenya/Tunisia (*F*_ST_ = 0.406); 13 in the comparison between the sub-populations MoroccoB and Northern Galilee (*F*_ST_ = 0.676); nine in the comparison between the sub-populations MoroccoB and Kenya/Tunisia (*F*_ST_ = 0.380).

## Discussion

The eight strains of *L*. *aethiopica*, four of which were isolated from human cases of LCL and four from human cases of DCL that came from different Ethiopian locations between 1972 and 1994, were typed successfully, using the set of 12 microsatellite markers developed and optimized for their species-specificity to the species *L*. *tropica* and previously applied in phylogenetic analyses of that species [28,29]. This in itself indicated a close genetic relationship between these two species, which was also shown by previous studies applying various molecular methods [16,18,19].

In the NJ tree (Fig 1) and network (Fig 2) and the Bayesian statistics (S1 Table), the eight strains of *L*. *aethiopica* grouped together closely and, as a group, were situated well within the cluster of African strains of *L*. *tropica*, which, incidentally, also contained all the strains of *L*. *tropica* from the focus north of the Sea of Galilee. However, by the FCA, they were seen as distinctly separate from all the strains of *L*. *tropica* (Fig 3 and S3 Fig). The Israeli and Palestinian strains of *L*. *tropica* and the one from the Sinai Peninsula, Egypt, formed a cluster clearly separated from the strains of *L*. *tropica* from the north, south and west African, the other Middle Eastern and the Indian foci in all four analyses. The strains of various geographical origins isolated between 1949 and 1999 together with the Turkish and Palestinian ones formed a second cluster (Figs 1–3). A third cluster suggested by the Bayesian clustering approach contained the eight strains of *L*. *aethiopica*, 20 African strains of *L*. *tropica* from Morocco, Tunisia, Kenya and Namibia, two Turkish ones and ten from the Israeli focus north of the Sea of Galilee [8]. The six Moroccan (Morocco A) and two Turkish strains of *L*. *tropica* in the sub-population MoroccoA/Turkey clustered more closely to the Turkish strains of the sub-population Turkey/old strains/Palestine than they did to the African strains as found by the NJ tree (Fig 1), the network (Fig 2) and the FCA results (Fig 3), which was in contrast to the Bayesian statistics. The population containing the strains of *L*. *aethiopica*, most of the African strains of *L*. *tropica*, and the strains of *L*. *tropica* from the northern Galilee displayed a high level of genetic variation. The trees' long branches and numerous cross connections between branches indicate long evolutionary distances and a high degree of genetic interconnection among these strains (Figs 1 and 2). A property of STRUCTURE is to put strains failing to get assigned to a genetically distinct population, into a separate one, which is what might have happened with the African strains of *L*. *tropica* and the Israeli ones from northern Galilee.

The separation of the African strains of *L*. *tropica* and the Israeli ones from northern Galilee into sub-populations was essentially very similar in all four analytical approaches. In both distance-based approaches, the NJ tree (Fig 1) and the network (Fig 2), the strains of *L*. *aethiopica* presented as a single compact group within the cluster of African strains of *L*. *tropica* and the Israeli ones from the focus north of the Sea of Galilee. Four other sub-populations of *L*. *tropica* were confirmed by all four approaches, each consisting of strains from specific geographical areas, correlating the genetic character of the leishmanial parasites with their geographical origins, which are essentially the geographical origins of their human hosts, animal reservoirs and sand fly vectors. The sub-population Kenya/Tunisia suggested by the Bayesian analysis was not confirmed by the other methods used and its strains were very paraphyletic in the NJ tree (Fig 1) and the network (Fig 2). Accordingly, they were distributed over a relatively wide area in the FCA (Fig 3 and S3 Fig). One Kenyan strain of *L*. *tropica*, MHOM/KE/1981/NLB162, clustered together with the four Namibian strains by the Bayesian statistics and in the NJ tree and the network, although it separated from the monophyletic group at an early point. This strain was clearly closer the other Kenyan strains of *L*. *tropica* in the FCA.

**Fig 4:**
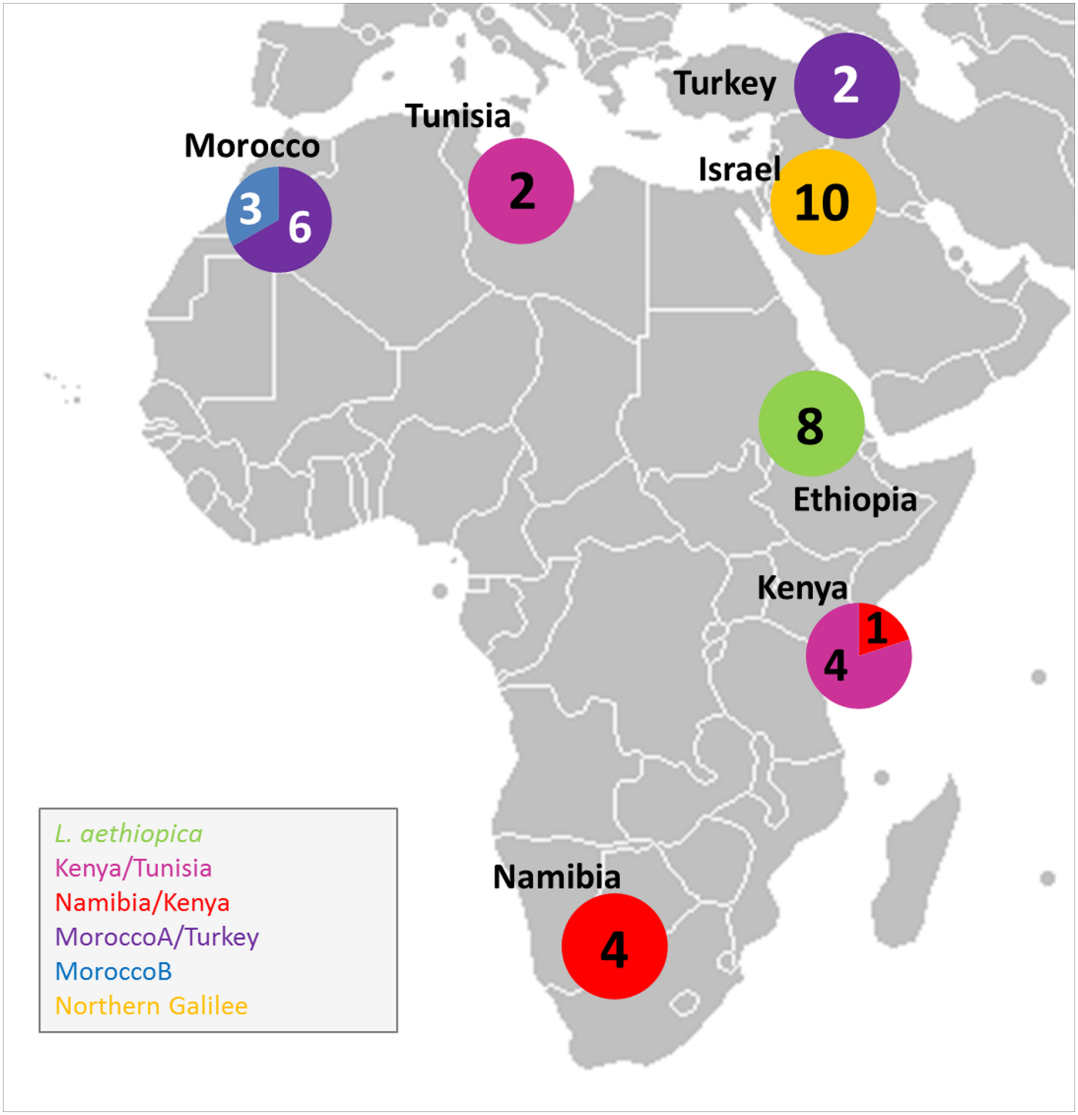
Map showing the geographical distribution of the six sub-populations in the main population Africa/Galilee. The six colours in the circles represent the six sub-populations where the colours correspond with those in Figs 1–3. The numbers in the circles indicate the number of strains of each sub-population from the country specified. Reprinted from and modified after http://commons.wikimedia.org/wiki/Maps_of_the_world#/media/File:BlankMap-World-v2.png under a CC BY license.

The map in Fig 4 shows the geographical distribution of the distinct sub-populations of the strains that clustered in the population Africa/Galilee, again, indicating the correlation between the genetic diversity of strains of *L*. *tropica* and their geographical origins as reported in previous phylogenetic studies on *L*. *tropica* [7,28,29]. This study indicated a relatively close genetic inter-relationship between the strains of *L*. *aethiopica* and those of *L*. *tropica*. How close either species is to *L*. *major* remains unanswered as microsatellite markers for *L*. *major* were not included. The microsatellite marker set developed for *L*. *major* [25]could be applied to *L*. *aethiopica* in future studies to reveal the genetic relationship between these two species. However, preliminary results of a single-nucleotide polymorphism (SNP) analysis, using data from whole genome sequencing (WGS) that included strains of *L*. *aethiopica*, *L*. *tropica* and *L*. *major*, indicated that *L*. *aethiopica* was genetically closer to *L*. *tropica* (Moser and Schönian, personal communication). The WGS-SNP analysis also supported the results of the FCA analysis presented here, which clearly separated the strains of the species *L*. *aethiopica* from those of the species *L*. *tropica* (Fig 3 and S3 Fig).

In a study of strains of *L*. *aethiopica* that included six of the eight strains of *L*. *aethiopica* studied here, the strains separated into two sub-groups [17]. No such separation of the eight strains of *L*. *aethiopica* occurred in this study although the NJ tree in Fig 1 does show the formation of two branches among the eight strains of *L*. *aethiopica*. However, this division of strains did not correlate with human clinical disease conditions, geographical distribution or time of isolation (S1 Table) and, generally, did not accord with the conclusions of citation [17]. Like the many other studies that used various targets, technologies and methods [10,11,17], the MLMT applied here did not distinguish strains of *L*. *aethiopica* that caused DCL from those that caused LCL. Other studies have also shown no correlation between microsatellite profiles and human clinical disease condition (CL and VL) [27,28]. None of this is surprising, since it has been considered for a long time that the progression and final outcome of infection depends on the immune status and capability of the people infected rather than on differences in the infectious agents [42]. What is seen in the case of *L*. *aethiopica* regarding CL, which is self-curing, and DCL, which is chronic and continuous without treatment, is like what is seen in the case of *L*. *tropica* regarding LCL, which is self-curing, and leishmaniasis recidivans (LR), which is chronic and continuous without treatment.

This study revealed a significant level of intra-species variation among the eight strains of *L*. *aethiopica* analysed, each of which had a different profile, which compares well with the study done by Le Blancq *et al*., who applied MLEE to 29 strains of *L*. *aethiopica* that encompassed 13 different zymodemes [11] and that done by Schonian *et al*., who applied ITS-RFLP [17]. The heterogeneity value (*H*_o_) of 0.266 for the population consisting of the eight strains of *L*. *aethiopica* was relatively high, compared to the populations consisting of strains of *L*. *tropica* that gave a mean *H*_o_ of 0.139 (Table 2). Also, the number of alleles was the highest among the strains of *L*. *aethiopica* (A = 3.250) despite the low sample number (n = 8). In addition, the long branches in the NJ tree and the network indicated quite large evolutionary distances between the single strains (Figs 1 and 2). In the network, the cross connections between the branches indicate many reticulation events like recombination, hybridisation or gene transfer within the sub-population consisting of the eight strains of *L*. *aethiopica* (Fig 2). In the FCA, the strains within this sub-population were very distantly separated from each other, more than were the strains of *L*. *tropica* within their respective clusters (Fig 3).

In summary, the ability of the complete microsatellite marker set developed for *L*. *tropica* to work also with strains of *L*. *aethiopica* was interesting, indicating a close genetic similarity between these two leishmanial species, particularly regarding the strains of *L*. *tropica* isolated in Africa, in some cases, in foci very distant from those supplying the strains of *L*. *aethiopica*. There was no correlation between microsatellite profiles and the type of leishmaniasis, LCL versus DCL, seen in the human cases.

## Supporting Information

S1 Fig Calculation of the most probable number of populations based on the results of Bayesian statistics. A, calculation including all the strains to determine the most probable number of main populations; B-D, sub-structuring of the main populations: B = Africa/Galilee, n = 40; C = Turkey/old strains/Palestine, n = 57; D = Israel/Palestine, n = 67 where n is the number of strains.
(PDF)

S2 Fig Strains of *L*. *tropica* from Morocco, Turkey and Namibia sharing membership in the genetic populations. The few strains that showed allele frequency patterns of two populations are magnified in the figure. The strains are coloured according to their subsequent assignment to a sub-population: red = Namibia/Kenya; purple = MoroccoA/Turkey; grey = Turkey/old strains/Palestine.
(PDF)

S3 Fig Factorial correspondence analysis of the strains of *L*. *aethiopica* and *L*. *tropica* in the population Africa/Galilee. The genetic distances calculated by FCA based on allele similarities and shown in a 3-dimensional space. Each square represents one genotype. The colours correspond to the results of Bayesian clustering. The software requires pre-assignment to single populations so that the strains were assigned to the six sub-populations proposed by STRUCTURE.
(PDF)

S1 Table The strains of *L*. *aethiopica* and *L*. *tropica* used in this study and their microsatellite profiles. The strains of *L*. *aethiopica* are given in bold typeface; the rest are strains of *L*. *tropica*. The WHO codes give information about the host of this specimen as well as the country and the year of its isolation. Detailed information about the origins of the strains is given in the column “geographic origin” where known. The column “source” specifies the host and, if mammalian, its form of the disease from which the samples were collected; CL = cutaneous leishmaniasis, VL = visceral leishmaniasis, LCL = local cutaneous leishmaniasis, DCL = diffuse cutaneous leishmaniasis. In the column “microsatellite profiles” the designations specify the species, *Laet* = *L*. *aethiopica* and *Ltro* = *L*. *tropica*, and the profile number, MS = microsatellite and a three digit number. Assignment to populations and sub-populations is according to the Bayesian statistics though slight differences were found with the other approaches used. The colours of sub-populations correspond with those in Figs 1–4. The column headed Doc, for documentation, gives the publication first citing the given strain's microsatellite profile: * = Schwenkenbecher et al., 2006 [7]; ** = Krayter et al., 2014 [28]; *** = typed during this study. The lengths of the fragments containing the microsatellites are given for each strain and each marker.
(XLSX)

S2 Table Estimation of the genetic distances between the sub-populations. The mean fixation indices (*F*_ST_) result from pairwise comparisons of the distinct sub-populations. Values are categorized into little (<0.05), moderate (0.05–0.15), great (0.15–0.25), and very great (>0.25) differentiation. Insignificant values are indicated by an asterisk (*).
(PDF)

## References

[1] Ashford RW, Bray MA, Hutchinson MP, Bray RS (1973) The epidemiology of cutaneous leishmaniasis in Ethiopia. Trans R Soc Trop Med Hyg 67: 568–601. 4150462 10.1016/0035-9203(73)90088-6

[2] Alvar J, Velez ID, Bern C, Herrero M, Desjeux P, Cano J, et al (2012) Leishmaniasis Worldwide and Global Estimates of Its Incidence. PLoS One 7: e35671 10.1371/journal.pone.0035671 22693548 PMC3365071

[3] Gebre-Michael T, Balkew M, Ali A, Ludovisi A, Gramiccia M (2004) The isolation of Leishmania tropica and L. aethiopica from Phlebotomus (Paraphlebotomus) species (Diptera: Psychodidae) in the Awash Valley, northeastern Ethiopia. Trans R Soc Trop Med Hyg 98: 64–70. 14702839 10.1016/s0035-9203(03)00008-7

[4] Chance ML, Schnur LF, Thomas SC, Peters W (1978) The biochemical and serological taxonomy of Leishmania from the Aethiopian zoogeographical region of Africa. Ann Trop Med Parasitol 72: 533–542. 736662 10.1080/00034983.1978.11719357

[5] Le Blancq SM, Cibulskis RE, Peters W (1986) Leishmania in the Old World: 5. Numerical analysis of isoenzyme data. Trans R Soc Trop Med Hyg 80: 517–524. 3810783 10.1016/0035-9203(86)90128-8

[6] Grove SS (1989) Leishmaniasis in South West Africa/Namibia to date. S Afr Med J 75: 290–292. 2648612

[7] Schwenkenbecher JM, Wirth T, Schnur LF, Jaffe CL, Schallig H, Al-Jawabreh A, et al (2006) Microsatellite analysis reveals genetic structure of Leishmania tropica. Int J Parasitol 36: 237–246. 16307745 10.1016/j.ijpara.2005.09.010

[8] Svobodova M, Votypka J, Peckova J, Dvorak V, Nasereddin A, Baneth G, et al (2006) Distinct transmission cycles of Leishmania tropica in 2 adjacent foci, Northern Israel. Emerg Infect Dis 12: 1860–1868. 17326936 10.3201/eid1212.060497 PMC3291354

[9] Bray RS, Ashford RW, Bray MA (1973) The parasite causing cutaneous leishmaniasis in Ethiopia. Trans R Soc Trop Med Hyg 67: 345–348. 4778189 10.1016/0035-9203(73)90111-9

[10] Schnur LF, Zuckerman A (1977) Leishmanial excreted factor (EF) serotypes in Sudan, Kenya and Ethiopia. Ann Trop Med Parasitol 71: 273–294. 921364 10.1080/00034983.1977.11687191

[11] Le Blancq SM, Belehu A, Peters W (1986) Leishmania in the Old World: 3. The distribution of L. aethiopica zymodemes. Trans R Soc Trop Med Hyg 80: 360–366. 3798530 10.1016/0035-9203(86)90318-4

[12] Rioux JA, Lanotte G, Serres E, Pratlong F, Bastien P, Perieres J (1990) Taxonomy of Leishmania. Use of isoenzymes. Suggestions for a new classification. Ann Parasitol Hum Comp 65: 111–125. 2080829 10.1051/parasite/1990653111

[13] Pratlong F, Lanotte G (1999) Identification, taxonomie et phylogénèse In: Dedet JP, editor. Les Leishmanioses. Paris: Ellipses pp. 22–40.

[14] Pratlong F, Dereure J, Ravel C, Lami P, Balard Y, Serres G, et al (2009) Geographical distribution and epidemiological features of Old World cutaneous leishmaniasis foci, based on the isoenzyme analysis of 1048 strains. Trop Med Int Health 14: 1071–1085. 10.1111/j.1365-3156.2009.02336.x 19624480

[15] Fraga J, Montalvo AM, Van der Auwera G, Maes I, Dujardin JC, Van der Auwera G (2013) Evolution and species discrimination according to the Leishmania heat-shock protein 20 gene. Infect Genet Evol 18: 229–237. 10.1016/j.meegid.2013.05.020 23722022

[16] Montalvo AM, Fraga J, Monzote L, Montano I, De Doncker S, Dujardin JC, et al (2010) Heat-shock protein 70 PCR-RFLP: a universal simple tool for Leishmania species discrimination in the New and Old World. Parasitology 137: 1159–1168. 10.1017/S0031182010000089 20441679

[17] Schonian G, Akuffo H, Lewin S, Maasho K, Nylen S, Pratlong F, et al (2000) Genetic variability within the species Leishmania aethiopica does not correlate with clinical variations of cutaneous leishmaniasis. Mol Biochem Parasitol 106: 239–248. 10699253 10.1016/s0166-6851(99)00216-9

[18] Nasereddin A, Bensoussan-Hermano E, Schonian G, Baneth G, Jaffe CL (2008) Molecular diagnosis of Old World cutaneous leishmaniasis and species identification by use of a reverse line blot hybridization assay. J Clin Microbiol 46: 2848–2855. 10.1128/JCM.00951-08 18614659 PMC2546705

[19] El Baidouri F, Diancourt L, Berry V, Chevenet F, Pratlong F, Marty P, et al (2013) Genetic structure and evolution of the Leishmania genus in Africa and Eurasia: what does MLSA tell us. PLoS Negl Trop Dis 7: e2255 10.1371/journal.pntd.0002255 23785530 PMC3681676

[20] Schonian G, Nasereddin A, Dinse N, Schweynoch C, Schallig HD, Presber W, et al (2003) PCR diagnosis and characterization of Leishmania in local and imported clinical samples. Diagn Microbiol Infect Dis 47: 349–358. 12967749 10.1016/s0732-8893(03)00093-2

[21] Talmi-Frank D, Nasereddin A, Schnur LF, Schonian G, Toz SO, Jaffe C, et al (2010) Detection and identification of old world Leishmania by high resolution melt analysis. PLoS Negl Trop Dis 4: e581 10.1371/journal.pntd.0000581 20069036 PMC2797090

[22] Kuru T, Janusz N, Gadisa E, Gedamu L, Aseffa A (2011) Leishmania aethiopica: development of specific and sensitive PCR diagnostic test. Exp Parasitol 128: 391–395. 10.1016/j.exppara.2011.05.006 21616071

[23] Kebede N, Oghumu S, Worku A, Hailu A, Varikuti S, Satoskar AR (2013) Multilocus microsatellite signature and identification of specific molecular markers for Leishmania aethiopica. Parasit Vectors 6: 160 10.1186/1756-3305-6-160 23734874 PMC3679749

[24] Kuhls K, Keilonat L, Ochsenreither S, Schaar M, Schweynoch C, Presber W, et al (2007) Multilocus microsatellite typing (MLMT) reveals genetically isolated populations between and within the main endemic regions of visceral leishmaniasis. Microbes Infect 9: 334–343. 17307010 10.1016/j.micinf.2006.12.009

[25] Al-Jawabreh A, Diezmann S, Muller M, Wirth T, Schnur LF, Strelkova MV, et al (2008) Identification of geographically distributed sub-populations of Leishmania (Leishmania) major by microsatellite analysis. BMC Evol Biol 8: 183 10.1186/1471-2148-8-183 18577226 PMC2447845

[26] Oddone R, Schweynoch C, Schonian G, de Sousa Cdos S, Cupolillo E, Espinosa D, et al (2009) Development of a multilocus microsatellite typing approach for discriminating strains of Leishmania (Viannia) species. J Clin Microbiol 47: 2818–2825. 10.1128/JCM.00645-09 19587302 PMC2738093

[27] Downing T, Stark O, Vanaerschot M, Imamura H, Sanders M, Decuypere S, et al (2012) Genome-wide SNP and microsatellite variation illuminate population-level epidemiology in the Leishmania donovani species complex. Infect Genet Evol 12: 149–159. 10.1016/j.meegid.2011.11.005 22119748 PMC3315668

[28] Krayter L, Bumb RA, Azmi K, Wuttke J, Malik MD, Schnur LF, et al (2014) Multilocus microsatellite typing reveals a genetic relationship but, also, genetic differences between Indian strains of Leishmania tropica causing cutaneous leishmaniasis and those causing visceral leishmaniasis. Parasit Vectors 7: 123 10.1186/1756-3305-7-123 24666968 PMC3987047

[29] Krayter L, Alam MZ, Rhajaoui M, Schnur LF, Schonian G (2014) Multilocus Microsatellite Typing reveals intra-focal genetic diversity among strains of Leishmania tropica in Chichaoua Province, Morocco. Infect Genet Evol 28: 233–239. 10.1016/j.meegid.2014.09.037 25308380

[30] Jamjoom MB, Ashford RW, Bates PA, Kemp SJ, Noyes HA (2002) Polymorphic microsatellite repeats are not conserved between Leishmania donovani and Leishmania major. Molecular Ecology Notes 2: 104–106.

[31] Jamjoom MB, Ashford RW, Bates PA, Kemp SJ, Noyes HA (2002) Towards a standard battery of microsatellite markers for the analysis of the Leishmania donovani complex. Ann Trop Med Parasitol 96: 265–270. 12061973 10.1179/000349802125000790

[32] Schwenkenbecher JM, Frohlich C, Gehre F, Schnur LF, Schonian G (2004) Evolution and conservation of microsatellite markers for Leishmania tropica. Infect Genet Evol 4: 99–105. 15157627 10.1016/j.meegid.2004.01.005

[33] Akuffo HO, Fehniger TE, Britton S (1988) Differential recognition of Leishmania aethiopica antigens by lymphocytes from patients with local and diffuse cutaneous leishmaniasis. Evidence for antigen-induced immune suppression. J Immunol 141: 2461–2466. 3171177

[34] Dieringer D, Schlötterer C (2003) Microsatellite analyser (MSA): a platform independent analysis tool for large microsatellite data sets. Molecular Ecology Notes 3: 167–169.

[35] Pritchard JK, Stephens M, Donnelly P (2000) Inference of population structure using multilocus genotype data. Genetics 155: 945–959. 10835412 10.1093/genetics/155.2.945 PMC1461096

[36] Evanno G, Regnaut S, Goudet J (2005) Detecting the number of clusters of individuals using the software STRUCTURE: a simulation study. Mol Ecol 14: 2611–2620. 15969739 10.1111/j.1365-294X.2005.02553.x

[37] Belkhir K, Borsa P, Chikhi L, Raufaste N, Bonhomme F (1996–2004) GENETIX 4.05, logiciel sous Windows TM pour la génétique des populations. Laboratoire Génome, Populations, Interactions, CNRS UMR 5000, Université de Montpellier II, Montpellier (France).

[38] Glaubitz JC (2004) CONVERT: A user-friendly program to reformat diploid genotypic data for commonly used population genetic software packages. Molecular Ecology Notes 4: 309–310.

[39] Tamura K, Peterson D, Peterson N, Stecher G, Nei M, Kumar S (2011) MEGA5: molecular evolutionary genetics analysis using maximum likelihood, evolutionary distance, and maximum parsimony methods. Mol Biol Evol 28: 2731–2739. 10.1093/molbev/msr121 21546353 PMC3203626

[40] Huson DH, Bryant D (2006) Application of phylogenetic networks in evolutionary studies. Mol Biol Evol 23: 254–267. 16221896 10.1093/molbev/msj030

[41] Lewis PO, Zaykin D (2001) Genetic Data Analysis: Computer program for the analysis of allelic data. 1.0 ed. http://hydrodictyon.eeb.uconn.edu/people/plewis/software.php.

[42] Turk JL, Belehu A (1974) Immunological spectra in infectious diseases Parasites in the Immunized host: Mechanisms of Survival. Amsterdam, London and New York: Associated Scientific Publishers pp. 101–122.

