# Supplementary figures and images for: The Genetic Relationship between Leishmania aethiopica and Leishmania tropica Revealed by Comparing Microsatellite Profiles
Source: PLoS One. 2015 Jul 21;10(7):e0131227. doi: 10.1371/journal.pone.0131227 (PMC4511230; doi:10.1371/journal.pone.0131227)

A

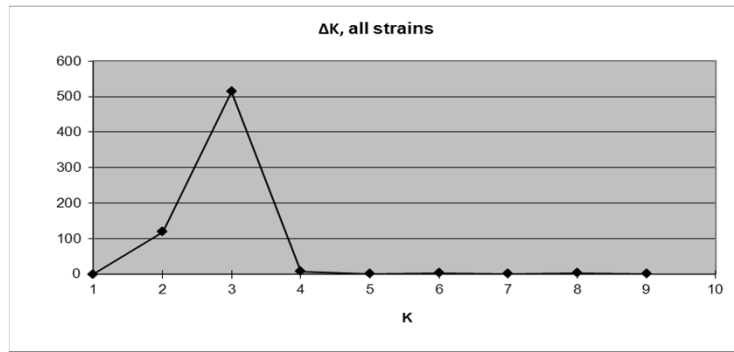

B

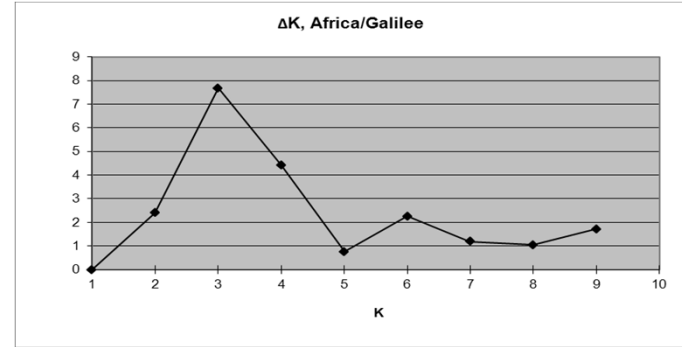

C

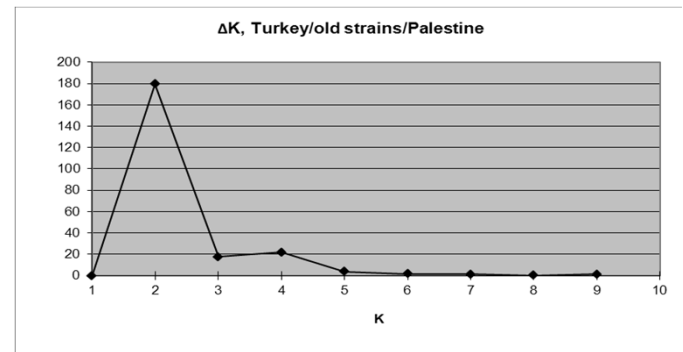

D

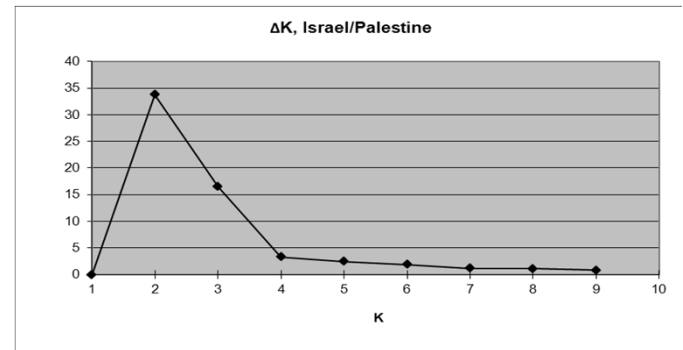

]

]

]

Supplement: S1 Fig — A, calculation including all the strains to determine the most probable number of main populations; B-D, sub-structuring of the main populations: B = Africa/Galilee, n = 40; C = Turkey/old strains/Palestine, n = 57; D = Israel/Palestine, n = 67 where n is the number of strains. (PDF) [file pone.0131227.s001.pdf]

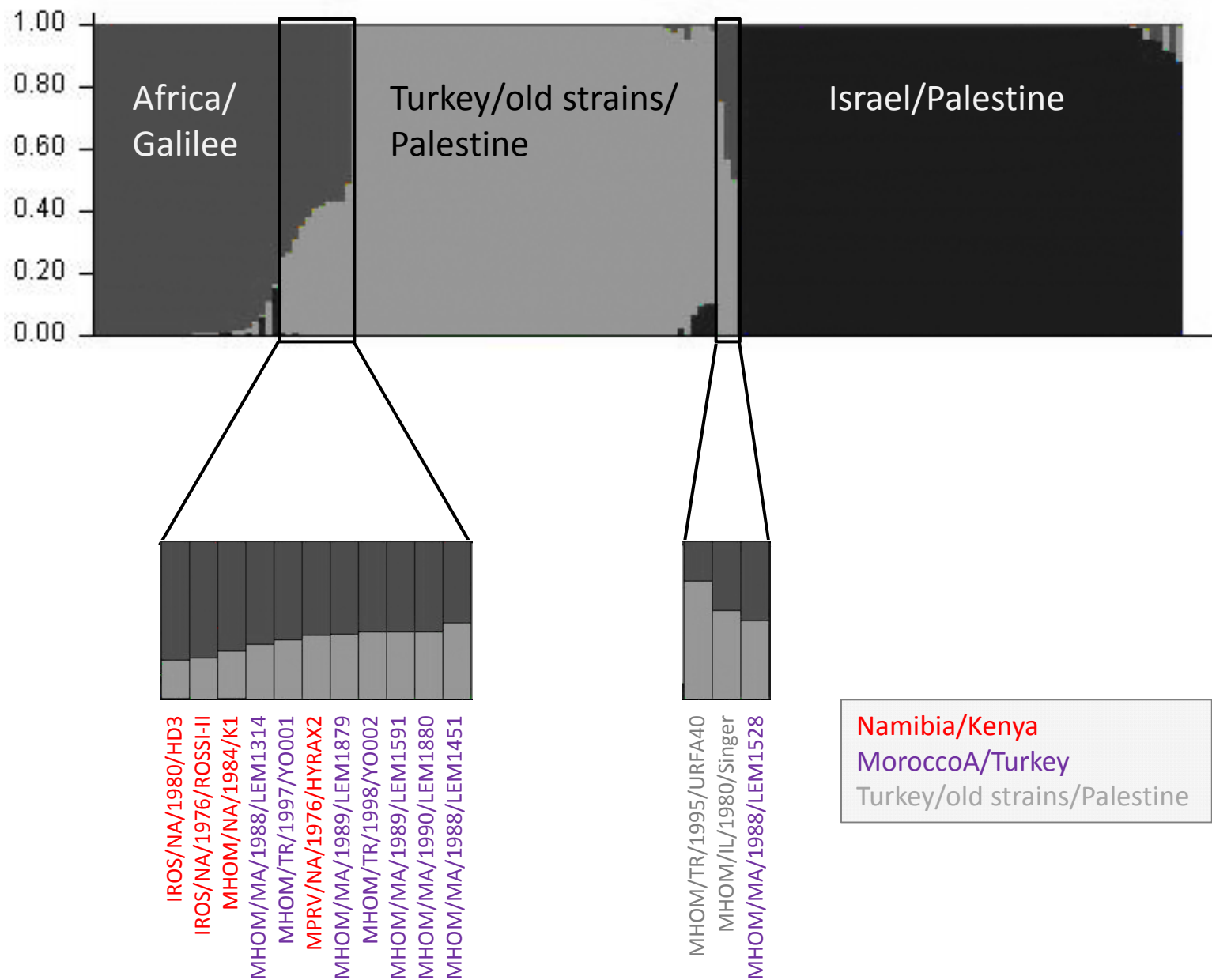

Supplement: S2 Fig — The few strains that showed allele frequency patterns of two populations are magnified in the figure. The strains are coloured according to their subsequent assignment to a sub-population: red = Namibia/Kenya; purple = MoroccoA/Turkey; grey = Turkey/old strains/Palestine. (PDF) [file pone.0131227.s002.pdf]

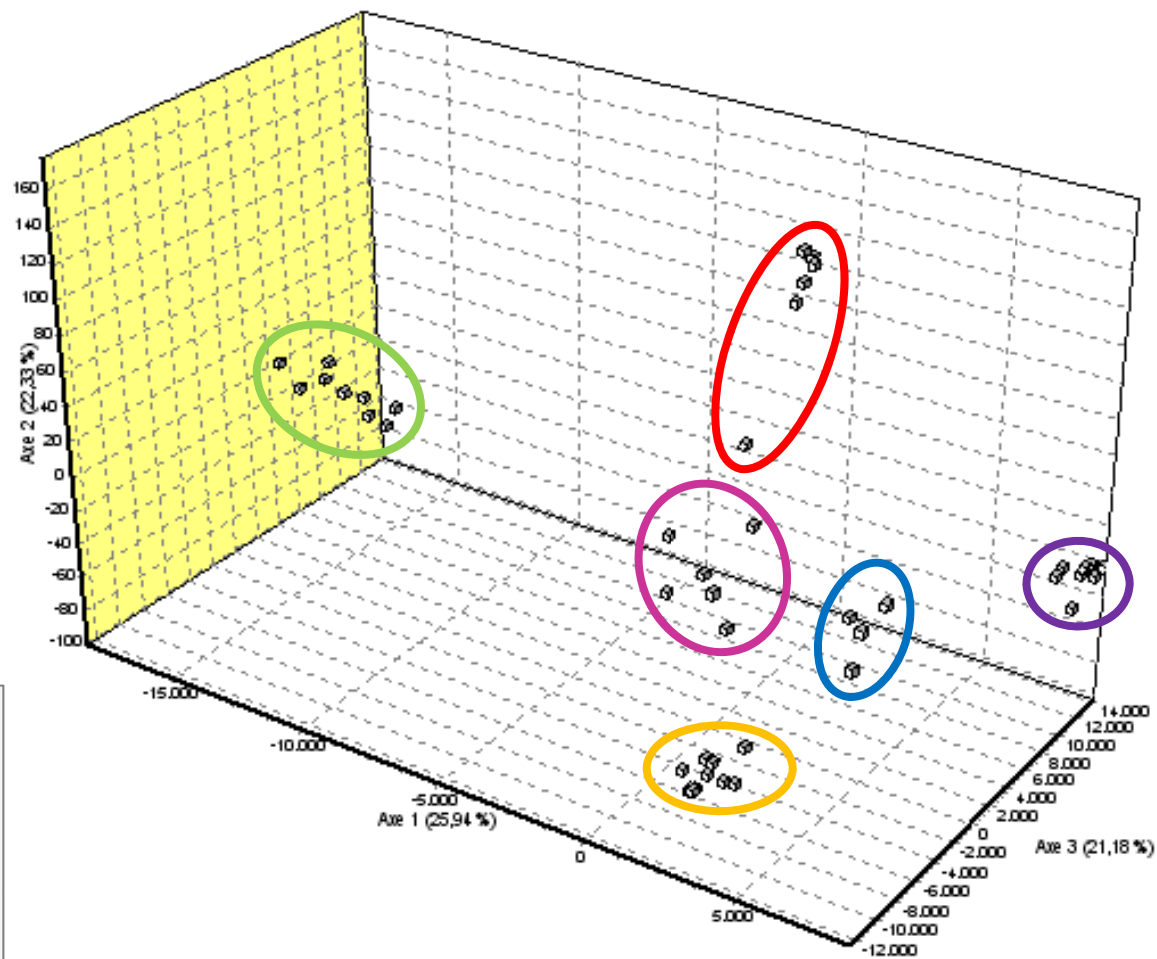

*L. aethiopica*  
Kenya/Tunisia  
Namibia/Kenya  
MoroccoA/Turkey  
MoroccoB  
Northern Galilee

Supplement: S3 Fig — The genetic distances calculated by FCA based on allele similarities and shown in a 3-dimensional space. Each square represents one genotype. The colours correspond to the results of Bayesian clustering. The software requires pre-assignment to single populations so that the strains were assigned to the six sub-populations proposed by STRUCTURE. (PDF) [file pone.0131227.s003.pdf]
