# Supplementary material for: The Genetic Relationship between Leishmania aethiopica and Leishmania tropica Revealed by Comparing Microsatellite Profiles
Source: PLoS One. 2015 Jul 21;10(7):e0131227. doi: 10.1371/journal.pone.0131227 (PMC4511230; doi:10.1371/journal.pone.0131227)
Supplement: S2 Table — The mean fixation indices (F ST) result from pairwise comparisons of the distinct sub-populations. Values are categorized into little (<0.05), moderate (0.05–0.15), great (0.15–0.25), and very great (>0.25) differentiation. Insignificant values are indicated by an asterisk (*). (PDF) [file pone.0131227.s005.pdf]

F_{ST}-values:	***L. aethiopica***	**Kenya/Tunisia**	**Namibia/Kenya**	**MoroccoA/Turkey**	**MoroccoB**	**Northern Galilee**	**Sanliurfa, TR**	**old strains/Palestine**	**Israel/Palestine**
L. aethiopica	0.000								
**Kenya/Tunisia**	0.306	0.000							
**Namibia/Kenya**	0.481	0.406*	0.000						
**MoroccoA/Turkey**	0.519	0.451	0.587	0.000					
**MoroccoB**	0.520*	0.380*	0.613	0.610*	0.000				
**Northern Galilee**	0.532	0.426	0.681	0.623	0.676*	0.000			
**Sanliurfa, TR**	0.698	0.638	0.699	0.612	0.773	0.772	0.000		
**old strains/Palestine**	0.534	0.418	0.534	0.385	0.578	0.596	0.287	0.000	
**Israel/Palestine**	0.755	0.658	0.778	0.745	0.823	0.759	0.793	0.688	0.000

<0.05	little genetic differentiation
0.05-0.15	moderate genetic differentiation
0.15-0.25	great genetic differentiation
>0.25	very great genetic differentiation

*not significant
